# Editorial: Venoms, Animal and Microbial Toxins

**DOI:** 10.3389/fphar.2021.706573

**Published:** 2021-05-26

**Authors:** Zhijian Cao, Jing-Lin Wang, Patrick Michael McNutt, Yuri N. Utkin, Delavar Shahbazzadeh, Heike Wulff, Hervé Kovacic, Jean-Marc Sabatier

**Affiliations:** ^1^ College of Life Sciences, Wuhan University, Wuhan, China; ^2^ Beijing Institute of Microbiology and Epidemiology, Beijing, China; ^3^ Wake Forest Institute for Regenerative Medicine, Winston-Salem, NC, United States; ^4^ Shemyakin-Ovchinnikov Institute of Bioorganic Chemistry RAS, Moscow, Russia; ^5^ Pasteur Institute of Iran (PII), Tehran, Iran; ^6^ Department of Pharmacology, University of California, Davis, Davis, CA, United States; ^7^ UMR7051 Institut de NeuroPhysiopathologie, Aix-Marseille Université, Marseille, France

**Keywords:** animal toxin, plant toxin, venom, bacterial toxin, viral toxin

The Research Topic titled “Venoms, Animal and Microbial Toxins” is focused on the structural and functional properties of animal derived venoms, plant and microbial toxins, as well as their molecular/cellular targets. It compiles seventeen key research articles and up-to-date reviews in the field to better describe venoms, the structural features of toxins (and derivatives thereof) and their various modes of action, through the analyses of their structural characteristics, structure-function relationship, and pharmacology.

Venomous animals such as scorpions, snakes, sea anemones, cone snails, worms, wasps, lizards and frogs, and microbes (e.g., bacteria, viruses and fungi) are the natural sources of diverse toxins to neutralize or kill their preys/hosts. These toxins (of various nature and size) are exhibiting a variety of modes of action by targeting ion channels, receptors, enzymes, neurotransmitter release, etc. Because of their potencies and wide range of bioactivities, researchers are actively studying toxins (and derivatives) focusing on their potential as candidate chemotherapeutic drugs (to treat pain, cancer, microbial infections, neurological and immune disorders) or as biological weapons (anthrax, ricin, conotoxins, etc.).

In this Research Topic, several articles are focused on the mode of action and/or synergy of toxins as well as venom compounds. For example, the review article by Ullah highlights snake venom L-amino acid oxidases (SV-LAAOs), which are enzymes catalyzing the stereospecific oxidation of L-amino acids to their corresponding α-keto acids. These key compounds are reportedly playing a role in many biological processes (apoptosis, platelet aggregation/inhibition, edema, hemorrhage and anticoagulation) and have been used as antimicrobials and anticancer agents. The author here describes the structure, mechanism of catalysis, and inhibition and substrate specificity of the characterized SV-LAAOs. Another article by Pucca et al. is focused on toxin synergism between phospholipases A_2_ (PLA_2_) and cytotoxins. The authors show how cytotoxins and PLA_2_ from distinct animal species (bees, vipers, elapids) can interact synergistically to enhance cell lysis. They further propose a mechanistic model of enhanced cell lysis by a synergistic action of both PLA_2_ and cytotoxins. The research article by Hess Lopes et al. reports on the action of *Loxosceles* spider venom sphingomyelinases D (SMases D) on lipid rafts and the activation of endogenous metalloproteinases from the ADAMs family. The authors found that SMases D alter lipid raft structures resulting in the activation of membrane bound proteases and subsequent proteolysis of cell surface proteins eventually leading to a pathology. The work by Xu et al. describes an action of the ricin toxin binding subunit B (RTB) on mouse macrophages. It was found to stimulate the production of TNF-α through activation of the transcription factor NF-κB via the TLR4 signaling pathway. Such work is important to better understand the mode of action of the highly toxic plant toxin ricin. Gigolaev et al. were able to switch the K^+^ channel subtype selectivity of the scorpion toxin MeKTx13-3 from the Asian scorpion *Mesobuthus eupeus* from the voltage-gated Kv1.1 to the Kv1.3 channel subtype using molecular modeling and mutagenesis. Such work helps to define the structural basis of toxin-to-ion channel recognition at the molecular level and illustrates the feasibility of redesigning venom peptides into potential biologic drug candidates since Kv1.3 constitutes an attractive target for immunosuppression. Similar applied work by Alvarado et al. reports on a “novel” insecticidal peptide against house crickets referred to as Osu1, from the venom of spider *Oculicosa supermirabilis*. Recombinantly expressed Osu1 was shown to potently modulate the human voltage-dependent Kv1.5 (hKv1.5) channel subtype, a potential target for atrial fibrillation therapy. Such a compound might serve as a lead to design structural analogs (and candidate drugs) with improved selectivity and/or potency toward this particular human K^+^ channel.

Other articles of the Research Topic are focused on the targets and fields of application of toxins or venoms. For example, bee venom-based acupuncture (BVA) is widely used in certain countries to treat a variety of disorders, including inflammatory and pain-related diseases. Adverse reactions such as anaphylaxis can occur. Lee et al. have examined the incidence rate of hypersensitivity reactions during or following BVA. The authors surveyed the medical records of 8,580 individuals treated by BVA (60,654 BVA treatments), and their potential clinical symptoms (allergy) were studied, highlighting a 0.047% incidence rate of anaphylaxis. An “*in-depth*” review article by de Castro Figueiredo Bordon et al., 2020 is also focusing on the various potential applications of toxins/venoms, from diagnostic tools to chemotherapeutic drugs. An overview of the current toxin-based marketed and candidate (non-marketed) drugs is presented. The advances and perspectives of candidate therapeutic molecules from scorpions, snakes, cone snails, sea anemones, spiders, hymenopterans, amphibians, and others marine and non-marine animals (leeches, bats, lizards, ticks, caterpillars, shrews), are critically discussed. An interesting report by Gao et al., 2021 compares the pancreatic damage induced by Paraquat (PQ) -a widely used herbicide in rats following two methods of PQ administration (intragastric infusion vs. intraperitoneal injection). The authors found that both methods could cause pancreatic damage, the most severe damage being associated with intragastric infusion. This study indicates that particular attention to the toxicity of PQ should therefore be observed by clinicians when this compound is ingested orally. The article by McArthur et al. highlights that, in addition to the voltage-gated Na_v_1.7 channel, the analgesic spider venom peptide Pn3a can also block high voltage-activated (HVA) calcium channels. This study reveals that L-, P/Q- and N-type Ca_v_ channels could be inhibited by Pn3a, contrary to R-type channel. Interestingly, the Pn3a inhibition of neuronal Ca_v_ currents is reportedly enhanced by opioid receptor activation.

The potential neutralization of the highly pathogenic bacterial toxins TcdA, TcdB and CDT (bacterium *Clostridioides difficile*) by human α-Defensin-5 is described in another article Korbmacher et al.. According to Korbmacher et al., α-defensin-5 should be considered as a candidate drug to treat severe associated human diseases such as pseudomembranous or fulminant colitis, and diarrhea. Thus, the beneficial role of α-defensin-5 would rely on its contribution in the first line host defense mechanism against microbes, as well as its inhibitory action against pathogenic bacterial toxins.

An important review article by Santos Menezes et al. deals with the voltage-gated sodium channelopathies and epilepsy. Indeed, voltage-gated sodium (Na_v_) channels are central to the action potential and mutation-induced dysfunction of Na_v_ channels may result in altered neuronal activity (e.g., epilepsy). Although Na_v_1.1 channels appear to be “key” regarding epilepsy, the authors provide an up-to-date overview on other epilepsy-related human Na_v_ channel subtypes (Nav1.1 to Nav1.3, Nav1.6 and Nav1.7).

Another key review article is provided by Osmakov et al.. The authors used animal, plant, and microbial toxins for the structural and functional/pharmacological characterization of membrane-associated acid-sensing ion channels (ASICs), which are sensors of extracellular pH variation involved in regulatory functions of neuronal and non-neuronal cells. The authors addressed the biophysical characteristics, architecture, natural ligands, structure-function relationships and therapeutic perspectives of ASICs.

Three articles of the Research Topic are dealing mainly with the structural aspects of toxins. The article by Ullah and Massod (2020) focuses on the structural features (amino acid sequences and 3D structures) of the poorly-studied snake venom phospholipases B, which are enzymes with the highest hemolytic potential in snake venoms. The authors are finally detailing their molecular model of phospholipase B from the snake *Bothrops moojeni*. The article by Câmara et al. describes a multiomics strategy for the identification, sequencing and preliminary screening of potentially bioactive peptides from the venom of tarantula spider *Acanthoscurria rondoniae*. Such multiomics strategies rely on proteomics, peptidomimetics and transcriptomics data coupled to some *in silico* predictions of antimicrobial and antitumor activities. The authors highlighted their “powerful” approach to discover new toxins in venoms and screen for their potential bioactivities.

The review article by Schmidt et al. is dealing with small, non-peptidic, molecules that are present in the venoms of cone snails in addition to conotoxins and conopeptides. The *Stephanoconus* clade of cone snails was mainly investigated because of its particular richness in small, non-peptidic compounds. So far, these molecules were found to be active on neurons and might be of interest in the potential treatment of specific neuronal disorders.

A last article authored by Jackson and Koludarov is particularly important in the field of toxinology, being centered on a complex but “key” question: how toxins actually got their toxic properties? The so-called “weaponization” of a molecule is reasonably and expectedly addressed by these authors.

Finally, in line with its main goal, this Research Topic clearly contributes to an “*in-depth*” knowledge of the animal/plant/microbial toxins (and derivatives) and their molecular targets expectedly opening the way to new attractive research in toxinology, toxicology and (neuro) pharmacology, in parallel to the design of new candidate drugs and/or appropriate antitoxin countermeasures. We strongly believe that this collection of articles exploring the complex world of toxins/toxic compounds and their targets, as well as venoms, will inspire many researchers and clinicians worldwide to continue working in or enter the exciting field of venoms and toxins.
